# Association between relative fat mass and colorectal cancer: a cross-sectional study

**DOI:** 10.3389/fnut.2025.1555435

**Published:** 2025-05-02

**Authors:** Yaping Wang, Shilong Song, Lu Zhang, Jingjing Zhang

**Affiliations:** ^1^Department of Medical Oncology, The First Affiliated Hospital of Bengbu Medical University, Bengbu, China; ^2^Department of Radiation Oncology, The First Affiliated Hospital of Bengbu Medical University, Bengbu, China

**Keywords:** relative fat mass, colorectal cancer, cross-sectional, NHANES, obesity

## Abstract

**Aims:**

We aimed to investigate the potential association between relative fat mass (RFM) and colorectal cancer (CRC).

**Design and methods:**

Data from the National Health and Nutrition Examination Survey (NHANES) spanning 1999 to 2020 were analyzed. Associations between RFM and CRC were analyzed using multiple logistic regression. Smoothed curve fitting was performed to conduct the association by sex. The stability of associations was assessed using subgroup analyses and interaction tests.

**Results:**

Overall, 52,281 participants over the age of 20 years were enrolled. The fully adjusted model observed a positive association between RFM and CRC, with one-unit increases in RFM linked to a 3% greater prevalence of CRC (OR = 1.03, 95%CI: 1.01, 1.06). A linear positive association was identified between RFM and CRC in male subjects, while a non-linear relationship was observed in females, with an inflection point at 42. Subgroup analysis revealed that age significantly modified the relationship between RFM and CRC (*P* for interaction = 0.0085).

**Conclusion:**

RFM is strongly associated with CRC prevalence in US adults. Further large-scale prospective investigations are warranted to for verification.

## 1 Introduction

Colorectal cancer (CRC) is a common malignancy, ranking third in terms of global incidence and second only to lung cancer in mortality, accounting for nearly one in ten cancer cases and deaths (1). Data from the USA indicate that despite annual reductions in CRC-associated deaths over the last decade the mortality rate for those under 50 years old is increasing, and prevention and treatment efforts remain challenging (2). The development of CRC is a slow and multistep process involving multiple risk factors, including genetics, consumption of a Western diet high in red, processed meat, and fats, intestinal flora, obesity—, especially visceral fat or abdominal obesity—, and metabolic syndrome (3, 4).

Obesity is defined primarily by an overabundance of adipose tissue. Adipose tissue, especially visceral adipose tissue (VAT), has been linked with a greater likelihood of developing cardiovascular disease (CVD), metabolic syndrome, and cancer (4, 5). Obesity also represents an important risk factor for several types of cancers, including CRC, with chronic low-grade inflammation and metabolic factors, such as adipokines and inflammatory cytokines, contributing mechanistically to this elevated risk (6, 7). Body mass index (BMI) is traditionally used as the primary metric for evaluating obesity. However, BMI has marked limitations, as it is unable to provide an accurate reflection of the distribution of body fat and shows poor sensitivity in assessing the relationship between obesity and CRC (8–10). In contrast, the newer index, relative fat mass (RFM), determined from height and waist circumference (WC) measurements, provides a superior estimation of the total percentage of body fat validated against dual-energy X-ray absorptiometry (DXA) (11). RFM is currently reported to be associated with several obesity-related conditions, including diabetes mellitus, metabolic syndrome (MetS), nonalcoholic fatty liver disease (NAFLD), and CVD (12–15). However, the relationship between RFM and CRC is still unknown.

This research utilized data from the National Health and Nutrition Examination Survey (NHANES) spanning 1999–2020 to investigate the association between RFM and CRC in the US population.

## 2 Methods

### 2.1 Study participants

NHANES is a nationally representative survey conducted by the National Center for Health Statistics (NCHS), collects data on diets, and laboratory measurements, physical examinations, interviews, and demographics of US citizens. All data were publicly available from the website, with the survey participants having provided informed consent.

**Figure 1:**
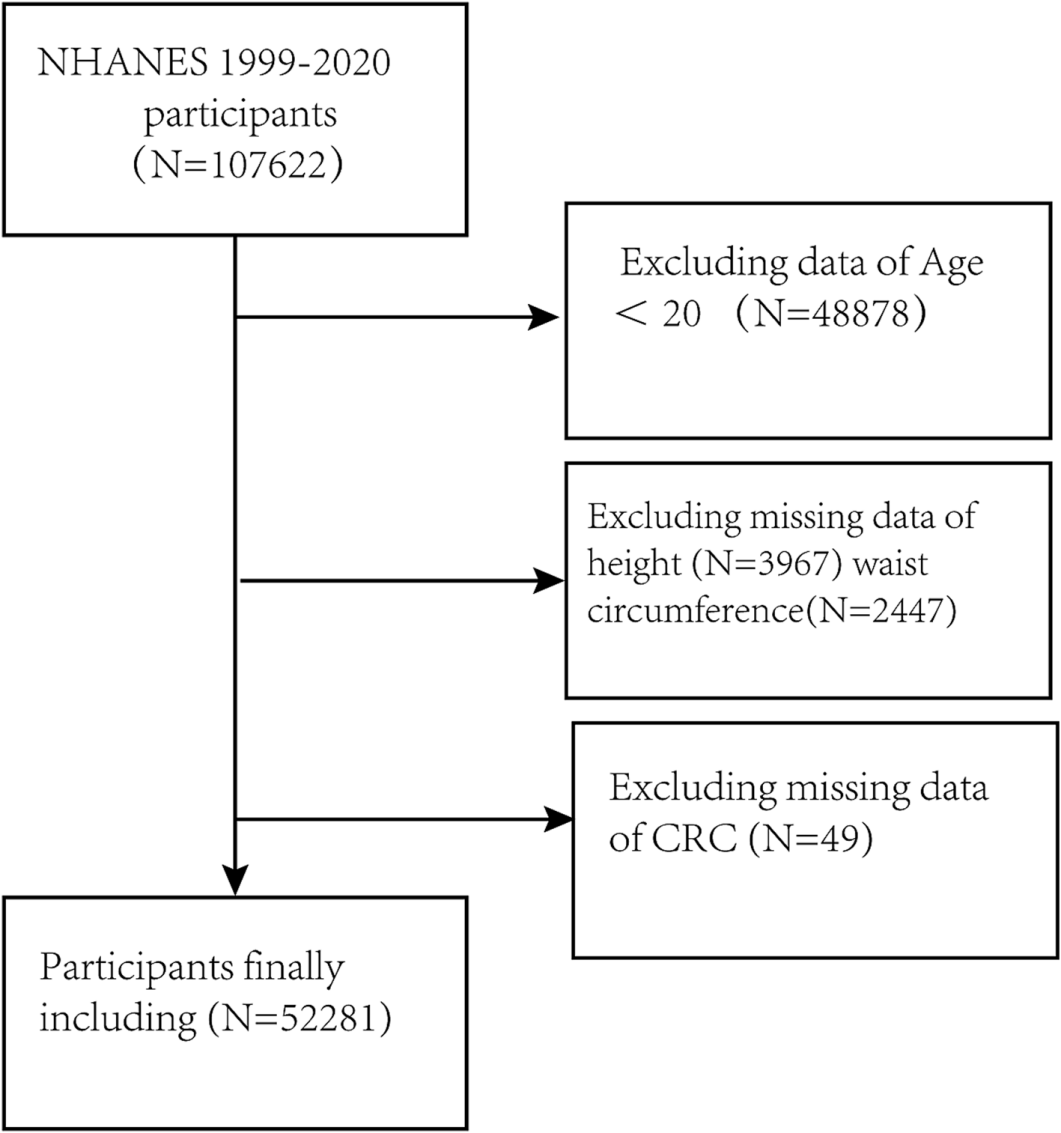
Flow chart of the study.

Here, the data of all participants in NHANES from 1999 to 2020 (*n* = 107,622) were evaluated. Individuals who were < 20 years of age or were missing clinically important data on height, WC, and CRC were excluded. The final enrollment was 52,281 participants. (Figure 1).

### 2.2 Definition of variables

The anthropometric measure RFM represented the primary exposure factor in this investigation; this assesses the level of obesity by measuring height and WC. The basic anthropometric measurements, including WC and height, were collected from participants by trained health technicians at Mobile Examination Center (MEC). RFM was determined as: RFM = 64 - (20 × height/WC) for men and RFM = 76 - (20 × height/ WC) for women (11).

The CRC diagnostic data were obtained through a structured questionnaire. Data on CRC patients were obtained through the ‘Ever told you had cancer or malignancy’ and ‘What kind of cancer’ items, which form part of the Medical Conditions Questionnaire (MCQ).

Based on previous studies and clinical significance (16, 17), this study analyzed covariates such as sex, age, race, educational attainment, and marital status. Smoking was defined as ‘smoking at least 100 cigarettes in life.’ Five classifications of alcohol consumption were recognized according to the amount consumed (18). The systemic immune-inflammation index (SII) was calculated using hematological parameters (19). Exercise was defined according to the questionnaire as ‘moderate activity over the past 30 days’ or ‘moderate work activity.’ Further details of the covariates are provided in Supplementary Table 1.

### 2.3 Statistical analyses

Descriptive analyses were first performed based on the characteristics of the participants. Continuous variables are shown as means ± standard deviation, with categorical variables as percentages. To investigate the differences between CRC and non-CRC groups, Kruskal-Wallis rank sum tests were used for continuous variables, and chi-square tests were applied for categorical variables. Multiple logistic regression was used to explore the relationship between RFM and CRC, with the construction of three statistical models, specifically, an unadjusted Model 1, Model 2 with adjustments for sex, age, race, and educational attainment, and Model 3 with further adjustments for marital status, smoking, drinking, physical activity, and SII, based on the covariates of Model 2. RFM was divided into quartiles, and the association between each quartile and CRC was analyzed, with trend analysis performed. Weighted regression analyses accounted for the complex survey design by the incorporation of Cluster IDs (SDMVPSU), Strata (SDMVSTRA), and sampling weights (WTMEC4YR for 1999–2002 cycles, WTMEC2YR for 2003–2016 cycles, WTMECPRP for 2017–2020 cycles), following the NHANES analytic guidelines. Smoothed curve fitting with a generalized additive model (GAM) was conducted to ascertain whether a nonlinear relationship exists between RFM and CRC. A recursive algorithm was first implemented to identify the inflection point in a nonlinear relationship, followed by the construction of a two-piece linear regression model. Finally, subgroup analyses and interaction tests were undertaken. In order to verify the robustness of the core results, we performed sensitivity analyses with multiple imputations for missing covariates. Missing covariates were imputed using the Multiple Imputation by Chained Equations (MICE) method via the R MI procedure. Five complete datasets were generated after 10 iterations performed to ensure chain stability, and multiple logistic regression was undertaken on each dataset. The effect values from each dataset were then pooled using Rubin’s rules (Supplementary Table 2). Data were analyzed with EmpowerStats and R (version 4.3.2), with *p* < 0.05 considered statistically significant.

## 3 Results

### 3.1 Characteristics of study participants

The study included the data of 107,622 participants. After the exclusion of subjects lacking the necessary data on WC, height, and CRC, 52,281 were ultimately enrolled (Figure 1). The study included 51,934 individuals without CRC and 347 participants with CRC. Table 1 presents the demographic details of the study population, categorized by the presence or absence of CRC. In the subgroups based on the presence of CRC, there were significant differences in RFM, age, race, educational attainment, marital status, smoking, drinking, and SII, while no significant differences were seen in sex and physical activity. Patients with CRC were more likely to be obese, older, and to have a history of smoking compared to those without a diagnosis of CRC.

**Table 1:** Participant characteristics by CRC in NHANES 1999–2020.

Characteristics	Non-CRC *N* = 51, 934	CRC *N* = 347	*p*-value
RFM, Mean ± SD	35.55 ± 8.67	37.30 ± 7.97	<0.001
Age, Mean ± SD	48.99 ± 17.78	69.60 ± 11.71	<0.001
Sex, *n* (%)			0.440
Male	25,112 (48.35)	175 (50.43)	
Female	26,822 (51.65)	172 (49.57)	
Race, *n* (%)			<0.001
Mexican American	8,957 (17.25)	23 (6.63)	
Other Hispanic	4,410 (8.49)	20 (5.76)	
Non-Hispanic White	22,491 (43.31)	213 (61.38)	
Non-Hispanic Black	11,162 (21.49)	73 (21.04)	
Other Race	4,914 (9.46)	18 (5.19)	
Education level, *n* (%)			0.007
Less than high school	13,558 (26.13)	114 (32.95)	
High school	12,062 (23.25)	83 (23.99)	
More than high school	26,263 (50.62)	149 (43.06)	
Marital status, *n* (%)			<0.001
Married/living with partner	31,141 (60.53)	179 (52.03)	
Widowed/divorced/separated	11,070 (21.52)	143 (41.57)	
Never married	9,240 (17.96)	22 (6.40)	
Smoke, *n* (%)			<0.001
Yes	23,484 (45.25)	206 (59.37)	
No	28,412 (54.75)	141 (40.63)	
Drinking, *n* (%)			<0.001
Never drinker	6,643 (14.15)	42 (13.46)	
Former drinker	7,739 (16.48)	94 (30.13)	
Light drinker	15,793 (33.63)	127 (40.71)	
Moderate drinker	7,222 (15.38)	24 (7.69)	
Heavy drinker	9,562 (20.36)	25 (8.01)	
Physical activity, *n* (%)			0.389
Yes	21,269 (41.37)	134 (39.07)	
No	30,148 (58.63)	209 (60.93)	
SII, Mean ± SD	556.92 ± 381.45	639.89 ± 407.45	<0.001

Among the 52,281 participants, the amount of missing values for the covariates was as follows: 52 (0.1%) for educational attainment, 486 (0.93%) for marital status, 38 (0.07%) for smoking, 5,010 (9.58%) for drinking, 521 (1%) for physical activity, and 2,197 (4.2%) for SII.

### 3.2 Association between RFM and CRC

Table 2 shows the relationship between RFM and CRC. The findings indicate that higher RFM is associated with an increased likelihood of CRC. Models 1, 2, and 3 all demonstrated positive associations between RFM and CRC, with odds ratio (OR) and 95% confidence interval (95%CI) of 1.02 (1.01, 1.04), 1.04 (1.01, 1.06), and 1.03 (1.01, 1.06), respectively. All *p*-values were below 0.05. In Model 3, when assessing groups Q2, Q3, and Q4 against the reference group Q1, the risk of CRC was significantly increased by 53, 95, and 85% (*P* for trend = 0.0317). Similar trends were apparent in Model 1 (*P* for trend = 0.0003) and Model 2 (*P* for trend = 0.0015). Weighted logistic regression further supported the robustness of the primary findings. (Supplementary Table 3).

**Table 2:** Association between RFM index and CRC.

Exposure	Crude model		Minimally adjusted model		Fully adjusted model	
	Model 1		Model 2		Model 3	
	OR (95%CI)	P-value	OR (95%CI)	P-value	OR (95%CI)	P-value
RFM	1.02 (1.01, 1.04)	0.0002	1.04 (1.01, 1.06)	0.0015	1.03 (1.01, 1.06)	0.0106
RFM quartile						
Q1 (7.756–29.152)	Ref		Ref		Ref	
Q2 (29.152–35.018)	2.09 (1.49, 2.94)	<0.0001	1.48 (1.04, 2.09)	0.0276	1.53 (1.04,2.25)	0.0290
Q3 (35.018–42.804)	1.93 (1.36, 2.72)	0.0002	2.05 (1.34, 3.14)	0.0009	1.95 (1.21, 3.14)	0.0058
Q4 (42.804–58.412)	2.09 (1.49, 2.94)	<0.0001	2.26 (1.36, 3.74)	0.0015	1.85 (1.06,3.23)	0.0315
*P* for trend		0.0003		0.0015		0.0317

Model 1: No covariates were adjusted.

Model 2: Adjusted for sex, age, race, and educational attainment.

Model 3: Adjusted for sex, age, race, educational attainment, marital status, smoking, drinking, physical activity, and SII.

**Figure 2:**
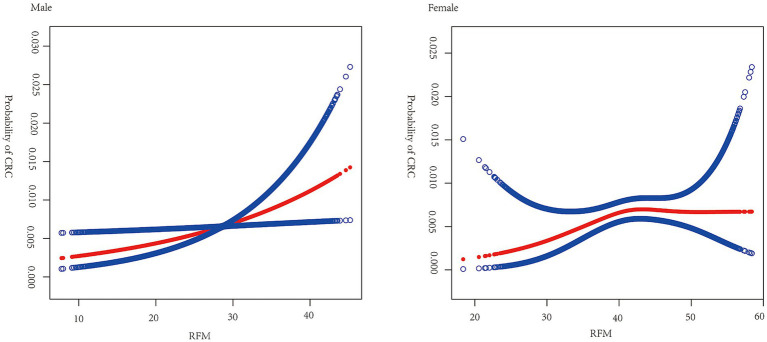
Smooth curve fitting for RFM and CRC by sex. Relationship between RFM and CRC was detected by the generalized additive model. Adjusted for age, race, educational attainment, marital status, smoking, drinking, physical activity, and SII.

Figure 2 shows the results of smoothed curve fitting between RFM and CRC by sex. In male participants, a positive relationship was identified between RFM and CRC, while in females, the association was nonlinear. An inflection point of 42 was found for RFM, with a positive association between RFM and CRC seen to the left of the point (OR = 1.09, 95% CI: 1.01, 1.19, *p* = 0.0363) and no significant association to the right of the point (OR = 0.97, 95% CI: 0.90, 1.03, *p* = 0.2973) (Table 3).

**Table 3:** Threshold effect analysis of RFM on CRC using a two-piecewise linear regression model in female participants.

Outcome	OR (95%CI)	*P*-value
Fitting by linear regression model	1.02 (0.98, 1.06)	0.2852
Fitting by two-piecewise linear regression model		
Inflection point (K)	42	
OR1 < K	1.09 (1.01, 1.19)	0.0363
OR2>K	0.97 (0.90, 1.03)	0.2973
OR2/OR1	0.88 (0.78, 1.00)	0.0553
Logarithmic likelihood ratio test *P*-value	0.048	

Adjusted for age, race, educational attainment, marital status, smoking, drinking, physical activity, and SII.

### 3.3 Subgroup analyses

**Figure 3:**
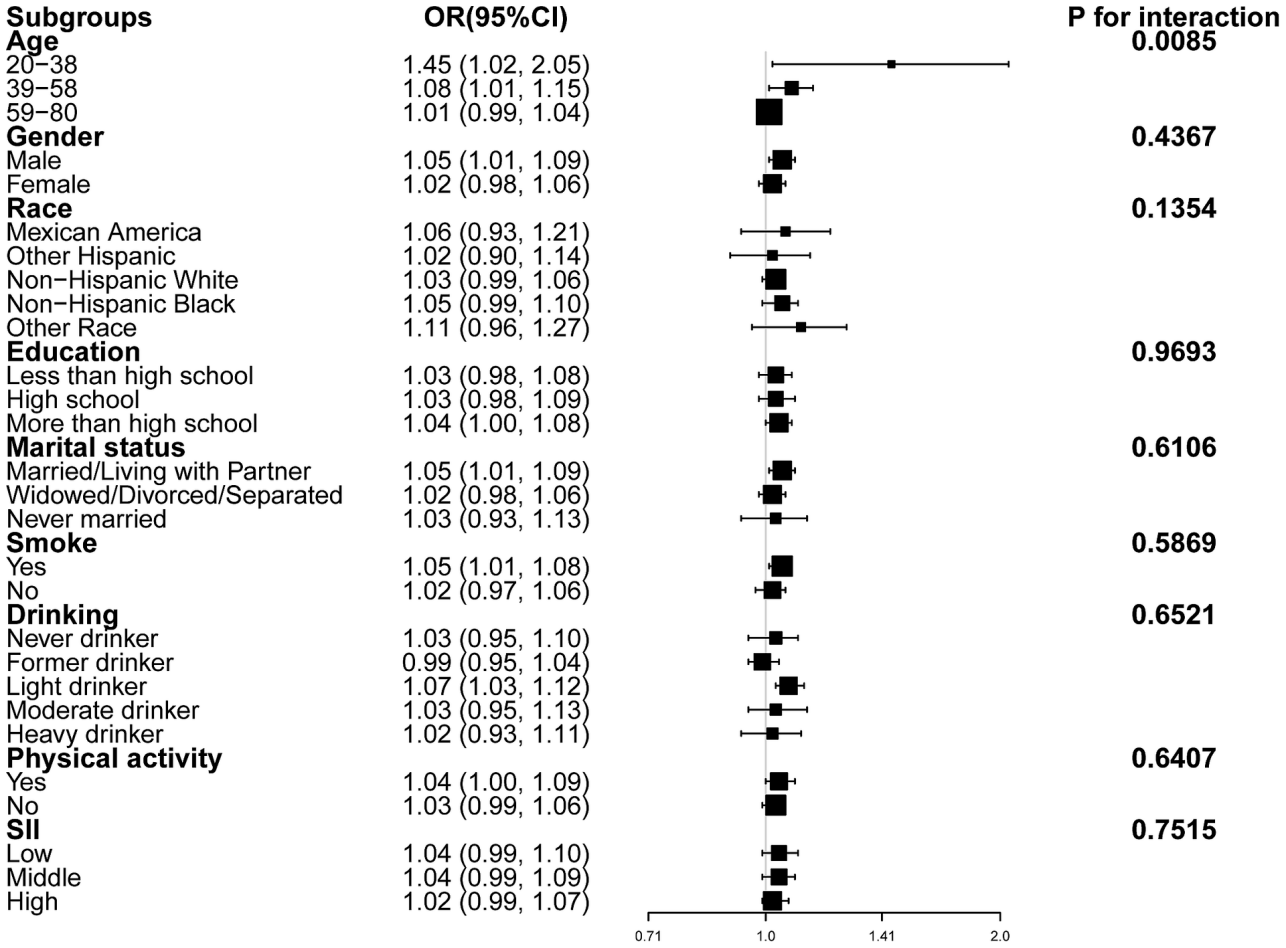
Forest plot of subgroup analyses of the relationship between RFM and CRC after adjusting all covariates except for the stratification variable itself.

Subgroup analyses and interaction tests were conducted across strata of sex, age, race, educational attainment, marital status, alcohol intake, smoking habit, physical activity, and SII (Figure 3), finding that only age significantly modified the association between RFM and CRC (*P* for interaction = 0.0085).

## 4 Discussion

The present cross-sectional analysis of 107,622 US adults aged ≥20 years found that RFM, analyzed as both a continuous and categorical variable, was positively associated with CRC. The relationship was linear in males but not in female subjects. In addition, subgroup analyses and interaction tests revealed a stronger association in participants aged 20–38 years, while the relationship remained consistent across other subgroups.

This appears to be the first investigation of an association between RFM and CRC. Recent studies have observed associations between RFM and a spectrum of chronic diseases, including diabetes, MetS, NAFLD, CVD, and lower urinary tract symptoms related to benign prostatic hyperplasia (LUTS/BPH) (12–15, 20). A retrospective cohort of 15,462 Japanese adults who were non-diabetic at baseline indicated a marked positive link between RFM and diabetes risk, especially in women (hazard ratio [HR]: 1.13, 95%CI: 1.04–1.24). The relationship was nonlinear. The RFM values for females and males were 39.23 and 23.08, respectively. Beyond these thresholds, the risk of diabetes was observed to increase substantially (12). A seven-year follow-up study in China reported that RFM was predictive of LUTS/BPH (20), while another cross-sectional study utilizing information from the Japanese NAGALA database observed a marked positive relationship between RFM and NAFLD, with nonlinear relationships observed in both males and females (15). Here, a strong association was found between RFM and CRC prevalence, aligning with previous research demonstrating significant associations between RFM and chronic diseases. Moreover, both the present study and two previous studies (12, 15) reported nonlinear relationships between RFM and disease outcomes. The difference is that our study identified a linear relationship in males and a non-linear relationship in females. The underlying reason for this difference remains unclear but could be attributed to sex-specific variations in fat distribution and hormonal profiles (21).

The potential mechanisms underlying the association between RFM and CRC remain unclear and may be related to the following mechanisms. Obesity, which is closely linked with elevated RFM metrics, has been extensively studied as a major risk factor for CRC. Obese individuals frequently exhibit marked insulin resistance and resultant hyperinsulinemia. Elevated insulin levels, coupled with raised levels of insulin-like growth factors (IGFs), can promote cellular proliferation and inhibit apoptosis, both of which are critical processes in CRC development (22). Pro-inflammatory cytokines released from visceral fat are also involved in the pathogenesis of numerous cancers, including colorectal tumors (23). Adipocyte-derived factors, notably adipokines such as leptin, which is typically elevated in obesity, have been linked to increased cell growth and invasion (24). Additionally, studies have indicated that obesity can drive cancer progression by inducing epigenetic changes in the colonic epithelium (25). Moreover, changes in sex hormone levels, which are linked to obesity, have been associated with a higher risk of CRC (26). In summary, RFM increases the probability of CRC development through multiple mechanisms, including metabolic dysfunction, chronic inflammation, epigenetic alterations, and hormonal imbalances.

This investigation has several notable strengths. First, it represents the first large-scale cross-sectional analysis assessing the association between RFM and CRC in the adult US population, using data from NHANES, a nationally representative dataset providing comprehensive health and nutritional information. Furthermore, multiple logistic regression models adjusted for a wide range of confounders, together with stratified analyses, were conducted to determine the links between RFM and CRC in different population subgroups. Despite these strengths, several limitations are present. First, the cross-sectional nature of the investigation does not permit the determination of causality. Second, the study did not account for all potential confounders, such as genetic predisposition, dietary patterns, which could influence both RFM and the prevalence of CRC. Third, CRC cases were identified solely on self-reported medical history rather than clinically validated diagnoses, and information on histopathological subtypes and tumor stages was not available. Finally, study participation was restricted to US adults, potentially limiting the generalizability of the results.

## 5 Conclusion

This study found that higher RFM is associated with an increased prevalence of CRC in adults. This relationship requires further verification in prospective cohort studies.

## Data Availability

Publicly available datasets were analyzed in this study. This data can be found at: https://wwwn.cdc.gov/nchs/nhanes/Default.aspx.
