# Supplementary material for: Association between relative fat mass and colorectal cancer: a cross-sectional study
Source: Front Nutr. 2025 May 2;12:1555435. doi: 10.3389/fnut.2025.1555435 (PMC12081260; doi:10.3389/fnut.2025.1555435)
Supplement: Supplementary file 1 [file Table_1.docx]

**Supplementary materials**

**Supplementary Table 1.** Detailed description and options of covariables in the NHANES database.

**Supplementary Table 2**. Association between RFM index and CRC in each of the five datasets after MI.

**Supplementary Table 3**. Association between RFM index and CRC. Weighted.

Supplementary Table 1. Detailed description and options of covariables in the NHANES database.

| Covariables | Description in NHANES |
| --- | --- |
| Age | Years |
| Race | Mexican American, other Hispanic, non-Hispanic White, non-Hispanic Black, other race |
| Educational attainment | Less than high school, high school, more than high school |
| Marital status | Married/living with partner, widowed/divorced/separated, never married |
| Smoke | Participants were asked whether they smoked at least 100 cigarettes in life (yes and no) |
| Drink | Never drinker (had < 12 drinks in a lifetime); former drinker (had ≥ 12 drinks in 1 year and did not drink last year, or did not drink last year but drank ≥ 12 drinks in a lifetime); current light drinker (≤ 1 drink per day for females, ≤ 2 drinks per day for males on average over the past year, or binge drinking [≥ 4 drinks/occasion for females, ≥ 5 drinks/occasion for males] on 1 day per month); current moderate drinker (≤ 2 drinks per day for females, ≤ 3 drinks per day for males, or binge drinking on 2 ~ 5 days per month); current heavy drinker (≥ 3 drinks per day for females, ≥ 4 drinks per day for males, or binge drinking ≥ 5 days per month). |
| SII | platelet count × neutrophil count/lymphocyte count |
| Physical activity | Participants were asked the questionnaire ‘moderate activity over the past 30 days’ or ‘moderate work activity’ (yes and no) |

Supplementary Table 2**:** Association between RFM index and CRC in each of the five datasets after MI.

|  | Crude model |  | Minimally adjusted model |  | Fully adjusted model |  |  |
| --- | --- | --- | --- | --- | --- | --- | --- |
|  | Model 1 |  | Model 2 |  | Model 3 |  |  |
|  | OR (95%CI) | P-value | OR (95%CI) | P-value | OR (95%CI) | P-value |  |
|  |  |  | Dataset 1 |  |  |  |  |
| RFM | 1.02 (1.01, 1.04) | 0.0002 | 1.04 (1.02, 1.06) | 0.0013 | 1.04 (1.01, 1.06) | 0.0014 |  |
| RFM quartile |  |  |  |  |  |  |  |
| Q1 | Ref |  | Ref |  | Ref |  |  |
| Q2 | 2.09(1.49,2.94) | <0.0001 | 1.49 (1.06, 2.11) | 0.0234 | 1.50 (1.06,2.13) | 0.0214 |  |
| Q3 | 1.93 (1.36, 2.72) | 0.0002 | 2.06 (1.35, 3.15) | 0.0008 | 2.06 (1.35, 3.15) | 0.0008 |  |
| Q4 | 2.09 (1.49, 2.94) | <0.0001 | 2.27 (1.37, 3.76) | 0.0014 | 2.27 (1.37,3.75) | 0.0014 |  |
| P for trend |  | 0.0003 |  | 0.0014 |  | 0.0014 |  |
|  |  |  | Dataset 2 |  |  |  |  |
| RFM | 1.02 (1.01, 1.04) | 0.0002 | 1.04 (1.02, 1.06) | 0.0013 | 1.04 (1.02, 1.06) | 0.0012 |  |
| RFM quartile |  |  |  |  |  |  |  |
| Q1 | Ref |  | Ref |  | Ref |  |  |
| Q2 | 2.09(1.49,2.94) | <0.0001 | 1.49 (1.06, 2.11) | 0.0233 | 1.51 (1.07,2.14) | 0.0198 |  |
| Q3 | 1.93 (1.36, 2.72) | 0.0002 | 2.06 (1.35, 3.15) | 0.0008 | 2.08 (1.36, 3.17) | 0.0007 |  |
| Q4 | 2.09 (1.49, 2.94) | <0.0001 | 2.27 (1.37, 3.76) | 0.0014 | 2.29 (1.39,3.80) | 0.0012 |  |
| P for trend |  | 0.0003 |  | 0.0014 |  | 0.0012 |  |
|  |  |  | Dataset 3 |  |  |  |  |
| RFM | 1.02 (1.01, 1.04) | 0.0002 | 1.04 (1.02, 1.06) | 0.0013 | 1.04 (1.02, 1.06) | 0.0012 |  |
| RFM quartile |  |  |  |  |  |  |  |
| Q1 | Ref |  | Ref |  | Ref |  |  |
| Q2 | 2.09(1.49,2.94) | <0.0001 | 1.49 (1.06, 2.11) | 0.0233 | 1.51 (1.07,2.14) | 0.0204 |  |
| Q3 | 1.93 (1.36, 2.72) | 0.0002 | 2.06 (1.35, 3.15) | 0.0009 | 2.07 (1.35, 3.16) | 0.0008 |  |
| Q4 | 2.09 (1.49, 2.94) | <0.0001 | 2.27 (1.37, 3.76) | 0.0015 | 2.29 (1.38,3.78) | 0.0013 |  |
| P for trend |  | 0.0003 |  | 0.0014 |  | 0.0012 |  |
|  |  |  | Dataset 4 |  |  |  |  |
| RFM | 1.02 (1.01, 1.04) | 0.0002 | 1.04 (1.02, 1.06) | 0.0013 | 1.04 (1.02, 1.06) | 0.0012 |  |
| RFM quartile |  |  |  |  |  |  |  |
| Q1 | Ref |  | Ref |  | Ref |  |  |
| Q2 | 2.09(1.49,2.94) | <0.0001 | 1.49 (1.06, 2.11) | 0.0234 | 1.51 (1.07,2.14) | 0.0198 |  |
| Q3 | 1.93 (1.36, 2.72) | 0.0002 | 2.06 (1.35, 3.15) | 0.0008 | 2.07 (1.36, 3.17) | 0.0007 |  |
| Q4 | 2.09 (1.49, 2.94) | <0.0001 | 2.27 (1.37, 3.76) | 0.0014 | 2.29 (1.38,3.78) | 0.0013 |  |
| P for trend |  | 0.0003 |  | 0.0014 |  | 0.0012 |  |
|  |  |  | Dataset 5 |  |  |  |  |
| RFM | 1.02 (1.01, 1.04) | 0.0002 | 1.04 (1.02, 1.06) | 0.0013 | 1.04 (1.02, 1.06) | 0.0012 |  |
| RFM quartile |  |  |  |  |  |  |  |
| Q1 | Ref |  | Ref |  | Ref |  |  |
| Q2 | 2.09(1.49,2.94) | <0.0001 | 1.49 (1.06, 2.11) | 0.0233 | 1.51 (1.07,2.14) | 0.0202 |  |
| Q3 | 1.93 (1.36, 2.72) | 0.0002 | 2.06 (1.35, 3.15) | 0.0009 | 2.07 (1.36, 3.17) | 0.0007 |  |
| Q4 | 2.09 (1.49, 2.94) | <0.0001 | 2.27 (1.37, 3.76) | 0.0015 | 2.29 (1.38,3.78) | 0.0013 |  |
| P for trend |  | 0.0003 |  | 0.0014 |  | 0.0012 |  |
|  |  |  | Combined effect values of five datasets |  |  |  |  |
| RFM | | 1.02 (1.00 1.03) | 0.0080 | 1.04 (1.02, 1.06) | <0.0001 | 1.04 (1.01, 1.06) | 0.0001 |
| RFM quartile | |  |  |  |  |  |  |
| Q1 | | Ref |  | Ref |  | Ref |  |
| Q2 | | 2.09(1.48,2.93) | <0.0001 | 1.49 (1.05, 2.10) | 0.0231 | 1.50 (1.06,2.13) | 0.0203 |
| Q3 | | 1.93 (1.36, 2.72) | 0.0002 | 2.06 (1.34, 3.14) | 0.0008 | 2.06 (1.35, 3.16) | 0.0007 |
| Q4 | | 2.09 (1.48, 2.93) | <0.0001 | 2.27 (1.37, 3.76) | 0.0014 | 2.28 (1.38,3.78) | 0.0012 |

Model 1: **No** covariates were adjusted.

Model 2: **Adjusted** for sex, age, race, and educational attainment.

Model 3: **Adjusted** for sex, age, race, educational attainment, marital status, smoking, drinking, physical activity, and SII.
Supplementary Table 3**:** Association between RFM index and CRC. Weighted.

|  | Crude model |  | Minimally adjusted model |  | Fully adjusted model |  |
| --- | --- | --- | --- | --- | --- | --- |
|  | Model 1 |  | Model 2 |  | Model 3 |  |
|  | OR (95%CI) | P-value | OR (95%CI) | P-value | OR (95%CI) | P-value |
| RFM | 1.03 (1.02, 1.05) | <0.0001 | 1.03 (0.99, 1.06) | 0.1254 | 1.02 (0.99, 1.06) | 0.1891 |
| RFM quartile |  |  |  |  |  |  |
| Q1 (7.756-29.152) | Ref |  | Ref |  | Ref |  |
| Q2 (29.152-35.018) | 2.19(1.39, 3.46) | 0.0010 | 1.50 (0.96, 2.34) | 0.0785 | 1.64 (1.03,2.59) | 0.0379 |
| Q3 (35.018-42.804) | 2.57 (1.57, 4.23) | 0.0003 | 2.08 (1.20, 3.61) | 0.0095 | 2.31 (1.30, 4.11) | 0.0049 |
| Q4 (42.804-58.412) | 2.38 (1.51, 3.47) | 0.0002 | 1.67 (0.89, 3.12) | 0.1109 | 1.57 (0.79,3.13) | 0.1975 |
| P for trend |  | <0.0001 |  | 0.1627 |  | 0.3222 |

Model 1: **No**covariates were adjusted.

Model 2: **Adjusted** for sex, age, race, and educational attainment.

Model 3: **Adjusted** for sex, age, race, educational attainment, marital status, smoking, drinking, physical activity, and SII.
